# Patient handoff from the perspective of nursing teams in hemodialysis units

**DOI:** 10.1590/0034-7167-2024-0301

**Published:** 2025-08-08

**Authors:** Cristina da Silva Fernandes, Dariane Verissímo de Araújo, Matheus Pinheiro Almeida, Nelson Miguel Galindo, Lívia Moreira Barros, Judith Sixsmith, Joselany Áfio Caetano

**Affiliations:** I Santa Casa de Misericórdia de Sobral. Sobral, Ceará, Brazil; II Universidade Federal do Ceará. Fortaleza, Ceará, Brazil; III Instituto Federal de Educação, Ciência e Tecnologia de Pernambuco. Recife, Pernambuco, Brazil; IV Universidade da Integração Internacional da Lusofonia Afro-Brasileira. Redenção, Ceará, Brazil; V University of Dundee. Dundee, Scotland, United Kingdom

**Keywords:** Nursing, Patient Safety, Communication, Patient Handoff, Hemodialysis Units, Hospital., Enfermagem, Segurança do Paciente, Comunicação, Transferência da Responsabilidade pelo Paciente, Unidades Hospitalares de Hemodiálise., Enfermería, Seguridad del Paciente, Comunicación, Pase de Guardia, Unidades de Hemodiálisis en Hospital.

## Abstract

**Objectives:**

to aimed to explore patient handoff practices among nursing teams in hemodialysis clinics.

**Methods:**

this study was conducted from April to June 2022, in six hemodialysis clinics in five health regions in Ceará, Brazil. The study involved 44 in-depth individual interviews with nursing professionals. Interviews occurred from April to June 2022 and were recorded for subsequent transcription.

**Results:**

four themes emerged from the data analysis: Information communicated during patient handoff; Failures in patient handoff; Communication-enabling technologies; and Nursing team’s perceptions on patient handoff.

**Final Considerations:**

the findings indicate that the patient handoff practices in hemodialysis clinics lack standardization and require improved communication among the nursing team. The lack of uniformity in information transmitted during shift handoffs poses a potential risk to patient safety and requires attention to enhance communication protocols.

## RESUMO

**Objetivos:**

explorar as práticas de transferência de pacientes entre as equipes de enfermagem em clínicas de hemodiálise.

**Métodos:**

o estudo foi realizado de abril a junho de 2022, em seis clínicas de hemodiálise em cinco regiões de saúde no Ceará, Brasil. O estudo envolveu 44 entrevistas individuais em profundidade com profissionais de enfermagem. As entrevistas ocorreram de abril a junho de 2022 e foram gravadas para posterior transcrição.

**Resultados:**

quatro temas emergiram da análise dos dados: Informações comunicadas durante a transferência de pacientes; Falhas na transferência de pacientes; Tecnologias facilitadoras da comunicação; e Percepções da equipe de enfermagem sobre a transferência de pacientes.

**Considerações Finais:**

os resultados indicam que as práticas de transferência de pacientes em clínicas de hemodiálise precisam de padronização e requerem melhor comunicação entre a equipe de enfermagem. A falta de uniformidade nas informações transmitidas durante as transferências de turno representa um risco em potencial para a segurança do paciente e requer atenção para aprimorar os protocolos de comunicação.

## RESUMEN

**Objetivos:**

explorar las prácticas de traspaso de pacientes entre los equipos de enfermería en las clínicas de hemodiálisis.

**Métodos:**

estudio realizado entre abril y junio de 2022 en seis clínicas de hemodiálisis de cinco regiones de salud de Ceará (Brasil). El estudio consistió en 44 entrevistas individuales en profundidad con profesionales de enfermería. Las entrevistas se realizaron entre abril y junio de 2022 y se grabaron para su posterior transcripción.

**Resultados:**

del análisis de los datos surgieron cuatro temas: Información comunicada durante el traspaso de pacientes; fallos en el traspaso de pacientes; tecnologías que facilitan la comunicación; y percepciones del equipo de enfermería sobre el traspaso de pacientes.

**Consideraciones Finales:**

los resultados indican que las prácticas de traspaso de pacientes en las clínicas de hemodiálisis son poco estandarizadas y requieren una mejor comunicación entre el equipo de enfermería. La falta de uniformidad en la información transmitida durante los traspasos de turno supone un riesgo potencial para la seguridad del paciente y requiere atención para mejorar los protocolos de comunicación.

## INTRODUCTION

Hemodialysis shift handoff entails intricate communication on chronic kidney disease profiles, treatment frequency, and advanced technology. Communication lapses among professionals during handoffs can lead to incidents and adverse events, risking patient outcomes. The frequency of handoffs and nurses attending to multiple patients necessitate extensive record-keeping^(1)^. Communication breakdowns, accounting for 70% of healthcare errors, significantly jeopardize patient safety^(2)^. These issues frequently stem from disorganized routines within healthcare teams, fostering impersonal communication environments^(3)^. A descriptive study in a private hospital’s hemodialysis service in southern Brazil correlated hypoglycemia cases with communication failures^(4)^. Moreover, in Goiania, Brazil, a cross-sectional study identified illegible hemodialysis prescriptions as the cause of 80% of communication failures^(5)^.

Communication is integral to the care process, particularly during shift handoffs between healthcare professionals. A 2021 systematic review emphasized that inadequate handoffs are linked to various risks, including information omissions, diagnostic errors, and treatment mistakes^(6)^. In hemodialysis clinics, transferring responsibilities between nursing teams is crucial due to treatment complexity, advanced technology use, and the need to manage patient profiles efficiently amid heavy workloads. In the US, a study showed that only 39% of nurses consider handoffs safe^(7)^. A study in Germany suggested that training is needed to improve communication; it found that safe communication training reduced preventable adverse events by 33.9%^(8)^.

Effective healthcare communication encompasses verbal exchanges, documentation, and handoffs. It must be objective, clear, and standardized to maintain crucial information for safe continuity of care^(9)^. Nurses, as team leaders and caregivers, are pivotal in ensuring patient safety during handoffs. This intricate process demands sufficient time and well-planned strategies, including education and support, to prevent omissions or inadequate communication that could endanger patients^(10)^.

Transferring patient responsibilities in hemodialysis clinics faces challenges due to lacking standardized tools and inadequate investment in ongoing education^(11)^. This can result in ineffective communication, risking patient safety. Identifying and addressing these flaws is crucial to ensure clear, standardized, and error-free information transfer, ultimately enhancing patient safety.

Implementing patient safety goals is one-way hospitals can adopt to improve the quality of their medical care. The six patient safety goals are: lowering the risk of infection associated with health measures; improving communication; increasing medicine safety; ensuring precise location, exact procedures, and exact patient during surgery; and lowering the risk of patient falls. Hospitals can evaluate patient safety goals and practices to identify and address safety issues that are relevant to day-to-day operations in hemodialysis units^(12)^.

Many adverse events occur due to the lack of optimal implementation of patient safety goals. The adoption of these goals in a hemodialysis unit is often considered less than ideal, as it is influenced by several factors, resulting in a high incidence of adverse events. Among the main causes are communication failures, both among healthcare professionals and between patients and their companions, which compromises patient safety. Furthermore, ineffective communication is noted to be one of the causes of 70% of errors committed in healthcare. This problem has been attributed to the daily routine of the healthcare team, as a disorganized routine contributes to transforming the care environment into a space in which communication becomes mechanized and impersonal^(13)^.

An in-depth investigation of the patient transfer process is needed to analyze communication patterns among nursing teams and reveal weaknesses in handoff practices that endanger patient safety. By exploring the theme and going through the steps to achieve the study objective, we seek to develop technological management tools that assist in the process of handover of the nursing team in hemodialysis clinics.

## OBJECTIVES

To explore patient handoff practices among nursing teams in hemodialysis clinics.

## METHODS

### Ethical Considerations

The study received approval from the Committee of Ethics in Human Research, aligning with ethical standards outlined in Brazilian Resolutions 466/12 and 510/16, safeguarding participants’ right to informed consent. His research was submitted for ethical assessment by the Federal University of Ceara and received an approved opinion on February 27, 2023. All participants were fully informed about the study’s purpose, procedures, and potential risks, and provided written consent before participation. Anonymity was ensured by identifying participants with the letters “NT” for nursing technicians and “N” for nurses, followed by interview sequence numbers. Participation was voluntary, and participants had the right to withdraw at any point without repercussions.

### Study type

Exploratory qualitative study. The study followed COREQ^(14)^ guidelines during design and execution.Consolidated Criteria for Reporting Qualitative research (COREQ) checklist for qualitative research. Regarding reflexivity, the researchers worked on collecting data in the field, in the critical analysis of statements associated with the interviewee’s behavior, in the critical analysis of the material collected to then make inferences about the results found.

### Study scenario, data source and methodological procedures.

Conducted from April to June 2022, it involved six hemodialysis clinics in five health regions of Ceará, Brazil, chosen for their high patient volume and diverse contexts. The sample was convenience-based, non-probabilistic, and non-random, drawn from available survey responses. The study included 44 nursing professionals (nurses and nursing technicians), who were selected based on their minimum of 6 months’ experience in hemodialysis clinics. Exclusion criteria included professionals not actively working during data collection.

### Data collection and organization

Data were collected through semi-structured interviews, where participants were provided with an interview script comprising sociodemographic and work-related questions, as well as inquiries regarding communication within the nursing team and shift handoff details. Additional questions covered safe care, patient safety training, and evaluations of communication and care safety levels. The instrument underwent evaluation and approval by three judges, experts in patient safety, to ensure clarity and relevance of questions. Data were collected at the participants’ workplace.

Two female lead authors, experienced qualitative researchers, specialist nurses, a doctoral student, and a master’s student, who were unfamiliar with the participants, conducted interviews in private rooms at hemodialysis clinics, with only the researcher and the participant present. After the study objectives were presented, participants were accommodated with minimal interruption. The interviews, which lasted approximately 30 minutes each, were audio-recorded on mobile phones and transcribed verbatim. Participants validated the information at the conclusion of the interview. No pilot testing was conducted, and interviews continued until theoretical data saturation was reached.

After writing the results and presenting them to the committee of the institution where the research originated, the final product of the study was sent via email to the coordinators of the research scenarios, as feedback on the discussions held during the investigation.

### Data analysis

The data were organized using IRaMuTeQ^®^ 0.7 Alpha 2.3.3.1 software, which employs Hierarchical Descendant Classification (HDC) analysis. HDC groups segments based on the chisquare test into classes displayed visually in a dendrogram created using IRaMuTeQ. This facilitated researchers’ access to contextually significant words for qualitative data analysis through interpretation^(15)^.

## RESULTS

### Characteristics of participants

Out of the 44 study participants, there were six nephrology specialist nurses and 38 nursing technicians. The majority of respondents were female (86%), with ages ranging from 20 to 51 years and an average age of 32 years. On average, participants had worked at the institution for 7.3 years. Regarding communication classification among the nursing team, 32 participants rated it as “good,” eight as “excellent,” and four as “reasonable.”

Following data analysis in IRaMuTeQ, four themes/classes were identified and labeled as follows: (Class 1) “Information communicated during patient handoff,” (Class 2) “Failures in patient handoff,” (Class 3) “Communication-enabling technologies,” and (Class 4) “Nursing team’s perceptions on patient handoff”.

### Class 1 - Information communicated during patient handoff

The communication among nurses during the transfer of patient responsibilities primarily occurs through the clinic’s shift report book, where relevant information from the shift, such as incidents and observations, is recorded.

*I’m gonna pass on all the incidents that happened. We register everything in the report book and verbally pass it on during the shift*. (N01)

*The morning nurse records things in her report book and informs me about the main stuff* [...] *the shift handoff is basically about what was most important during the day, in that shift.* (N02)

*The handoff is more about incidents, pending issues, what happened during the shift*. (N04)

*The shift handoff here is done through the report book and verbally. When the nurse arrives, we go through the pending tasks and any incidents*. (N05)

The nurses reported that it is not feasible to perform the shift handoff individually for each client, as approximately 40 patients are entrusted to a single professional.

*We don’t go bed by bed, if we were to do that, there would be 40 patients* [...]*. We deal with priorities*. (N01)

*We don’t go patient by patient, that’s not how nurses do it.* (N04)

One of the participating nurses in the study announced that the shift handoff takes place via phone call, given that the professionals work at the clinic on alternating days and adhere to a 12-hour work routine.

*The schedule is split between two nurses, with one working on Monday, Wednesday, and Friday for the full shift, and the other on Thursday, Friday, and Saturday. During the shift handoff, if there’s any complication, we usually talk on the phone*. (N03)

Most nursing technicians stated that they perform the transfer of care individually for each patient.

*I go through each patient bed by bed*. (NT03)

[...] *I talk about each patient*. (NT36)

One of the main vital signs communicated by healthcare professionals, especially nursing technicians, during the shift handoff is blood pressure (BP).

[...] *when she arrives, we would have already checked this blood pressure here, then we inform her, arrived with this pressure, but it’s already at this pressure now*. (NT01)

[...] *the pressure when he arrived and the pressure he has at the moment*. (NT05)

*The BPs that we check, if it’s already time to check the second-hour pressure, we hand over all of them, explain everything*. (NT09)

[...] *the initial blood pressure and the last blood pressure*. (NT14)

*Some of them already come in with low blood pressure, so we have to keep an eye on the pressure* [...] *we tell them if we’ve already checked the pressure and how the pressure is*. (NT2)

The participants stated that they also communicated about any occurrences and interventions during their shift handoff.

*If there was any incident, we share what the patient felt, if they mentioned feeling unwell or had hypotension*. (NT01)

*If any patient needed to be referred or had severe hypotension*. (N02)

*I share if the patient experienced any hypertensive spikes, if there’s a change in blood glucose levels, and other signs that can be observed and reported by them*. (NT23)

*If the patient encounters any issues during the time we take care of him, we have to pass on all the details meticulously to the next colleague receiving the shift*. (NT34)

Another important data in hemodialysis renal replacement therapy is weight, which must be assessed before and after each session. Moreover, this information is used to calculate the dry weight (ideal hydration level weight) and, thus, determine the most suitable therapeutic approach for the patient.

*I mention the dry weight they arrived with*. (NT09)

*If there was an increase or decrease in weight*. (NT16)

*When I arrive, my colleague tells me about the patient’s condition, the amount of weight they’re removing*. (NT20)

*We have to keep an eye on* [...] *the patient’s weight, at least here in hemodialysis, we work a lot with the patient’s weight*. (NT29)

The professionals also pass on information about prescribed medications, including those that have already been administered, those for home use, and those used in the hemodialysis system.

[...] *the medications they need to take, like Epogen, others have Ferumoxytol*. (NT03)

[...] *if there’s someone who will use antibiotics, Ferumoxytol, those who take them at home*. (N06)

*On my shift, I always share about the patients, if they’re on heparin, if there’s any bleeding, we ask for heparin to be stopped, and then we pass this on to the colleague to avoid the risk of blood clotting*. (NT32)

[...] *if they’re on heparin or not on heparin*. (NT33)

Nurse technicians affirmed that they provide all the necessary data to the professional taking over the care, including information required for programming the hemodialysis machine, such as the ultrafiltration rate (UF) and dialysis time.

*I mention the UF rate on the machine* [...]. (NT04)

[…] *what he’s losing on the machine*. (NT05)

[…] *informs the programmed weight, the programmed time*. (NT06)

Though Class 1, we explored specific details shared about information communicated during patient handoff and how they are conveyed, shedding light on the critical elements of effective communication in patient handoff. Class 2 investigates the various shortcomings and challenges that can occur during the patient handoff process.

### Class 2 - Failures in patient handoff

The participants mentioned failures related to the prescribed medications for the patients during the hemodialysis session.

*Even yesterday, I was on duty, and when I arrived, there was a patient without heparin, and the technician who came for the second shift didn’t receive this communication about the patient without heparin*. (NT28)

The participants also reported that they forget important data that needs to be communicated during the transfer of care, primarily due to the lack of standardization regarding what should be conveyed and the insufficient time available for the shift handoff.

*Some days we can’t pass on the information before the patients from the second shift come in; we can’t have such smooth communication*. (N06)

*At times, we forget; sometimes, the shift is hectic, and we forget to pass on something. This happens more when the shift is busier, maybe there’s an incident, or there are fewer staff members, making the shift more chaotic*. (NT31)

The nursing professionals mentioned that the patients have been under the care of the same nursing team for a considerable period, leading to the belief that it is unnecessary to communicate all information during the transfer of care, particularly patient identification data.

*We already know the patients, so it’s very familiar, as every day it’s the same people, we already have a lot of contact with them*. (NT24)

*We don’t need to pass on because they are simple things, and they are the same things*. (NT23)

Class 2 examined the instances where communication breaks down, misunderstandings arise, or critical information is omitted, providing insight into the areas where improvements are needed to enhance patient safety. Class 3 explores the role of technology in facilitating patient handoffs.

### Class 3 - Communication-enabling technologies

The nurses and nursing technicians in the study emphasized the efficiency of using radios for communication, especially when physically distant from each other, such as during patient transfers. Radios enable quick and convenient information exchange, complemented by telephone availability.

*We use radios, and it’s something that makes things easier*. (N02)

*The radio helps a lot*. (NT10)

*The radio is quite helpful because we can’t be at the door calling out, and everything is a bit distant, so we use the radio and the telephone*. (NT12)

*The radio is very useful, especially when the doctor or nurse is far away; the radio itself is quite helpful*. (NT14)

*We have radios and telephones here to give updates, and it’s good*. (NT18)

Two hemodialysis clinics have implemented an individual patient identification system, which consists of plates placed at each patient’s bedside, displaying information such as name, gender, age, prescribed medications, and patient risks.

*We have these tags that need to be identified, and we always leave them labeled for the next shift, just like the one there, it says ‘no heparin’ because my patient isn’t getting heparin. We need to be careful about the system’s clotting*. (NT29)

In one of the settings, the nurse reported that the team utilizes social media platforms such as WhatsApp to facilitate communication among the nursing staff.

*We even have a WhatsApp group, and when something happens, like when a patient needs a blood sample to be sent tomorrow morning, we record it here, but we also put it in the group so that everyone is aware*. (N01)

The participants affirmed that they use medical records for recording and tracking the information communicated during the transfer of care.

*Each patient has their own sheet* [...] *everything you do with the patient, any medication, you have to write it down, any doubts she has, she can just come and check*. (NT01)

*We have access to the patient’s medical records, and then we pass it on*. (NT02)

*We have the patient’s sheet, which includes the medication, any incidents, and any medication we gave them, anything like that*. (NT05)

The nursing teams reported that standardizing the data to be communicated during the transfer of care would prevent patient safety failures.

*There’s a hospital where they use the situation-background-assessment-recommendation* [SBAR] *technique. It’s a simple sheet that includes incidents, what’s needed, and what was done during the shift, very straightforward, very concise. It’s something I don’t see here; the handoff is purely oral, there’s nothing written. I think having something written for the handoff itself would be good because, in practice, we only remember what we can*. (N04)

*It would be great to have something with a more detailed handoff; it would be much better*. (NT16)

Class 3 delved into the use of communication tools, electronic health records, and other technological advancements that supported the transfer of patient information, highlighting their impact on the efficiency and quality of handoffs. Class 4 examines the perceptions and experiences of nursing teams.

### Class 4 - Nursing team’s perceptions on patient handoff

The participants affirmed the need for improvement in communication among the nursing team during the transfer of patient responsibility.

*Working with human beings is complicated, so sometimes, when we gather, we don’t communicate properly, and misunderstandings happen*. (NT17)

*It’s always good to improve communication*. (NT25)

Nursing professionals reported that simultaneous involvement in multiple tasks by their colleagues impedes communication during patient handoffs. This challenge arises from the limited time available to convey essential information effectively.

*Communication is good, but when a colleague has another job, it can be a bit challenging*. (NT21)

*I think here, the issue of punctuality should be improved. Some people arrive late, and sometimes, we pass on the shift to just one person, which ends up being detrimental because if something happens, that person claims they weren’t informed, even though it was communicated to others*. (NT26)

*Sometimes, there’s no one in the room to pass on the shift, so we have to wait for the next shift to come in. Sometimes, we end up being late for the next shift, and we can’t leave the room with patients alone; there always needs to be someone*. (NT27)

*I think paying attention is crucial because some colleagues are in a hurry to leave, and others are in a rush to come in, which can lead to communication gaps, and sometimes, they don’t receive the handoff*. (T28)

*Sometimes, a few colleagues arrive a little later, and it can be disruptive because we might forget something*. (NT30)

The quotes gathered in Class 4 uncovered how healthcare professionals viewed the patient handoff process, their attitudes, concerns, and suggestions, ultimately providing a deeper understanding of the human element in this critical aspect of healthcare delivery.

## DISCUSSION

The study findings reveal various issues with nurse handoffs in hemodialysis clinics, impacting treatment and patient outcomes. These include incomplete communication of vital information like blood pressure, weight, incidents, and machine settings; failures in medication adherence and omission of crucial data; and varied communication methods, including technology use such as social media, phones, and radios in certain clinics.

Our study emphasizes the significance of monitoring blood pressure during hemodialysis and underscores its critical role in patient handoffs. Previous literature has demonstrated the impact of hemodialysis on patients’ blood pressure^(16)^. For instance, research conducted in Thessaloniki, Greece, established a notable link between heightened blood pressure variability during hemodialysis and cardiovascular events in chronic kidney disease patients^(17)^. Communicating accurate blood pressure information during handoffs is essential to averting medical errors and improving patient outcomes.

During shift changes, the nursing team exchanges patient weight data, encompassing pre-hemodialysis, dry weight, and post-hemodialysis measurements. Research from Sweden has correlated interdialytic weight gain with cardiac issues and elevated mortality rates among hemodialysis patients^(18)^. Precise determination of dry weight is paramount for optimizing dialysis, reducing cardiovascular strain, and enhancing survival rates among individuals with chronic kidney disease^(19)^. Hence, ensuring the accurate transmission of verified weight information is crucial for the safety of dialysis patients.

During patient handoff, the nursing team communicates any encountered issues during hemodialysis. Dialysis patients frequently experience complications such as intradialytic hypotension, muscle cramps, nausea, and fatigue, significantly impacting their quality of life (as shown in a case series study in Turkey)^(20)^. Although serious complications like gas embolism or vascular access bleeding are rare, they can increase mortality risks^(21)^. Sharing these session events is vital for patient safety and enhancing prognosis.

During shift changes, the nursing team discusses hemodialysis machine settings, focusing on the ultrafiltration (UF) rate, crucial for patient survival. High weight-adjusted UF rates can lower blood pressure and increase cardiovascular mortality risk^(22)^. Interestingly, a study also found that obese individuals have a protective lower UF rate per weight compared to those with lower weight^(23)^. Hence, accurate communication of data for machine settings is vital to enhance care quality. However, lapses in communicating these settings during handoffs pose risks to patients, underscoring the need for precise communication to tailor settings and improve care quality.

Medication information is routinely exchanged during patient handoffs. A cross-sectional study in Jordan revealed that a majority of hemodialysis patients encountered medication-related issues, which escalated with age and number of medications^(24)^. This underscores the importance of vigilance when discussing prescriptions and medication administration for dialysis patients, given the high incidence of treatment-related problems in this vulnerable population.

Nursing professionals noted frequent forgetfulness during shift handoffs, attributing it to a lack of data standardization. An integrative review of 13 studies in 2021 revealed that a nursing handoff checklist ensures standardized performance, enhancing continuity of care and patient safety^(1)^. This highlights the potential of communication tools like checklists to improve the quality of care in hemodialysis.

The study highlights technology’s critical role in improving care team communication, emphasizing the use of radios and social media. Mobile technologies streamline tasks for nurses, stressing the need for investing in communication resources to prevent adverse events in hemodialysis clinics^(25)^. Participants recognize the urgent need for better communication within nursing teams. Literature also underscores improved communication among healthcare professionals to reduce adverse events and enhance care quality.

This study benefits from extensive geographic coverage, spanning six clinics across five diverse regions, enhancing the representativeness and generalizability of the findings. Additionally, the relatively large sample size enriches the dataset’s diversity, capturing various perspectives and enhancing the depth and breadth of qualitative analysis. However, limitations include relatively short interview durations, potentially compromising data depth and detail. Conducting research in a workplace setting may introduce biases or conversational constraints, warranting careful consideration of its impact. A randomized clinical trial in Barcelona, Spain, demonstrated that training nurses through role-playing enhances information sharing among the team and increases awareness of their roles and responsibilities^(26)^.

### Strengths and Limitations of the Work

One limitation of this study is the exclusion of one health region in Ceará. However, data from participants in other scenarios accurately depict patient handoff in hemodialysis clinics across the state.

### Recommendations for Further Research

The results of this study can strengthen the replication of further research on patient safety in dialysis, enriching the knowledge base and proposing tailored care strategies for diverse contexts nationwide. Additionally, these findings can inform the advancement of management technologies utilized in the care team’s handoff process.

### Implications for policy and practice

Communication is a fundamental aspect among the nursing team and for patient safety in hemodialysis units. However, there are communication failures among the nursing team regarding patient information in hemodialysis, considering that the information relayed consists of complications and vital signs, mainly blood pressure. Other information such as medications is often forgotten to be communicated, mainly due to the lack of standardized instruments for information transfer. Therefore, it is necessary to incorporate into clinical practice the use of communication means that strengthen patient safety and ensure the transfer of all information among healthcare professionals.

## FINAL CONSIDERATIONS

In the examined clinical settings, shift handoff communication predominantly utilizes printed materials and verbal exchanges. Nonetheless, apprehensions regarding medication safety persist due to the inherent difficulties associated with forgetfulness, multitasking, busy work environments, and rapid shift turnovers. Effective communication during handoffs is facilitated by adequate time allocation for meticulous exchange and the utilization of technology to streamline the transmission of information. These observations emphasize the imperative for standardized handoff protocols and comprehensive training initiatives aimed at enhancing patient safety and optimizing communication within care teams.

## Data Availability

Not applicable.
